# ‘Little Baby’s gone to Heaven’: A Mixed-Methods Study of Black Children’s Survival Disadvantage in Jim Crow-Era Arkansas

**DOI:** 10.1017/ssh.2025.15

**Published:** 2025-06-03

**Authors:** Cheryl Elman, Kathryn Feltey, Barbara Wittman, Corey Stevens, Molly B. Isenberg

**Affiliations:** 1 Social Science Research Institute, Duke University, Durham, NC, USA; 2 Department of Sociology, The University of Akron, Akron, OH, USA; 3 Lucy Cavendish College, University of Cambridge, Cambridge, UK; 4 Department of Sociology, Southern Illinois University-Edwardsville, Edwardsville, IL, USA; 5 Coordinator of Research and Evaluation, Chicago Police Department, Chicago, IL, USA

**Keywords:** Child mortality, racial health disparities, Jim Crow South, mixed methods, complete count IPUMS

## Abstract

Nearly all US Black children born before 1910 were born in the American South. We use a mixed-methods design to examine Black children’s survival disadvantage over the twentieth century’s turn under the rising regime of Jim Crow. We focus on 1910 Arkansas, taking advantage of within-state heterogeneity in agriculture (plantation vs. subsistence farming), disease environments, and geographic racial concentration (macro-segregation). This one-state focus allows purposive sampling of Works Progress Administration and Behind the Veil oral interviews of Arkansan Black Americans who were born or lived under the state’s Jim Crow regime. We also use the 1910 complete-count Integrated Public Use Microdata Series (IPUMS) linked to US Decennial and 1916 Plantation Censuses to examine race-related differences in child mortality rates among ever-married, parous Arkansas women (*n* = 234,811). Count regression models find the Black-White child mortality gap widest among Arkansas mothers economically tied to plantation vs. subsistence agriculture; exposed to worse health environments; living in tenant farm vs. owned-farm households; and with limited individual resources such as literacy. Oral accounts illustrate how Black children’s lives reflected contextual, living standard, psychosocial, and other health risks associated with the racialized policies and practices of the Jim Crow South; they capture otherwise hidden historical processes that linked the era’s institutional racism and child mortality.

## Introduction

Nearly two recorded centuries of higher Black than White child mortality rates testify to the long historical arm of US health disparities associated with race. Under Enslavement fewer than two of three Black children, measured in 1850–1860 data, survived to age 10 (Jones 2010: 34). At the twentieth century’s turn under Jim Crow (late 1870s–1950s), Black children’s mortality rates were about 58 percent higher than those of White children (Preston and Haines 1984). Civil Rights-era policies of the 1960s partly ameliorated mortality disadvantage (Almond et al. 2006) but between then and the twenty-first century’s turn, Black children’s death rates were higher than White children’s rates and were highest in the South (Singh 2010). Black children’s mortality disadvantage has persisted across southern institutional regimes (Omi and Winant 2015). It is important to examine their life conditions within each.

We focus on the life conditions of Black children in the early Jim Crow-era South. Most Black Americans, before and after the Civil War, lived in the South. Prior to 1900, over 90 percent did so, falling to about 78 percent in 1940 (Hobbs and Stoops 2002). Moreover, most southern Black Americans in 1910 lived in a rural corridor of counties devoted to plantation agriculture (U.S. Census Bureau 1916). Southern plantation agriculture before the Civil War was rooted in geographic and ecological conditions – involving climate, water, soil (Wright 2006) – and an enslaved labor supply (Woodman 1979). While Enslavement ended after the Civil War, plantation agriculture persisted as southern states institutionalized household tenancy systems to buttress rural labor supplies (Woodman 1995). By the twentieth century’s turn, plantation productivity – and much southern wealth – depended on hand labor^1^ of primarily-Black tenant households (Jung 2020; Woodman 2001). Tenant women and children generated one-third of southern plantation production in 1910 (Brannen 1924). Our first Aim is to examine the association between Black children’s survival disadvantage and their exposure to plantation agriculture. We do this by linking a 1916 US Plantation Census – fielded alongside but separate from the 13th (1910) US Decennial Census – to the complete-count 1910 IPUMS.

While we are concerned with Black children’s mortality risk in the context of plantation agriculture, we are as concerned about overlapping risks for southern children associated with being “Black” in the era of Jim Crow. Laws and practices assigned race by skin color and ancestry to (en)force physical and social separation in residence and institutions of education, property ownership, and occupation (Chafe et al. 1990). Racialized institutions perpetuated segregation and false ideologies about Black American inferiority to the 1950s and beyond (Darity and Mullen 2020). Southern Black children experienced racial discrimination – a fundamental cause of (poor) health production – from birth (Phelan and Link 2015; Williams and Mohammed 2013). Our second aim is to investigate the associations between plantation agriculture and Black children’s mortality risk in the larger context of the Jim Crow South. As Census data are less useful for this purpose, we draw qualitative samples from two sources: Federal Writers Project (FWP) interviews of formerly enslaved Black women (Works Project Administration 1936–1938) and Behind the Veil (BTV) interviews of Black persons born in Jim Crow states (Chafe et al. 1990). Interviewees lived through one or more southern pre-Civil Rights regimes, Enslavement, and Jim Crow. Their narratives illuminate ways in which living standards and exposure to Jim Crow-era institutions shaped Black children’s survival chances.

Qualitative samples are small, compared to complete-count IPUMS samples. We pursue a one-state analysis of Arkansas to allow for adequate sampling size of oral histories by geographic place, focusing on Arkansas for several reasons. First, it was both a BTV state and the most heavily sampled FWP state with the least “grave” data shortcomings (Cantrell 2004: 49). Second, urbanization and fertility decline (especially among White women) varied across southern states (Tolnay 1995). Arkansas, a postwar Trans-Mississippi Frontier,^2^ was a rural state with high Black and White fertility rates.^3^ Third, Arkansas’ three subregions allow exploration of early-twentieth-century contextual factors associated with Black children’s survival, including racial segregation, disease prevalence, and tenant farm prevalence (Franklin and Wilson 2020; Karbeah and Hacker 2023; Tolnay 1984).

We add to the literature by exploring child mortality in the context of plantation agriculture. Studies using data derived from US Decennial Censuses, alone, cannot address this: Decennial Censuses never enumerated plantations. Additionally, oral accounts provide exceptionally fine-grained descriptions of Black children’s life conditions. To preview findings, statistical analyses reveal a higher rate of child loss among Black compared to White Arkansas mothers and a wider child-mortality gap – adjusting for health environments and tenant farm prevalence – among mothers in plantation-holding counties. Oral accounts detail Black children’s health risks amidst plantations’ material surroundings and interconnected Jim Crow-era institutions, including schools and medical care.

We proceed as follows. We review prior studies and then provide historical background in three sections describing, more generally for the South while centering on Arkansas: (1) the turn-of-the-twentieth-century (re)institutionalization of plantation agriculture; (2) the plantation as a high-disease environment; and (3) plantation (vs. non-plantation) agriculture and socioeconomic position on an Agricultural Ladder. These sections generate hypotheses about plantation agriculture and Black children’s survival disadvantage. The Data and Methods sections follow. The first of two Results sections statistically evaluates hypotheses; the second draws from oral accounts to explore plantation conditions, life under Jim Crow, and Black children’s health and survival risk. Concluding remarks are in the Discussion.

## Prior studies

Most historical studies of US child mortality estimate national-level patterns using US Census and Vital Statistics data (see Hacker et al. 2023). They find race- and place-based factors to be major determinants of the Black-White gap. Mortality decline among White children was proceeding “at a moderate pace” by 1900, while mortality decline among Black children only took root after 1910 (Preston and Haines 1984: 85, 272). Black compared to White children’s mortality around 1910 was higher in rural (Karbeah and Hacker 2023; Logan and Parman 2018; Preston and Haines 1984) and urban places, across city size (Karbeah and Hacker 2023; Preston and Haines 1984).

Yet, the relationships between race, place, and child mortality were complex. Urban mortality decline commenced in the Northeast as White children’s health improved (Preston and Haines 1991) and lagged in the South as Black children’s high rates persisted (Feigenbaum et al. 2019). Black children had lower mortality rates (1900 data) in the 10 largest US cities compared to smaller US cities (Preston and Haines 1984) even as White children (Dribe et al. 2020) and Black and White children (Karbeah and Hacker 2023) suffered high mortality rates in rural county clusters within Appalachian and Mississippi River Valley states, including Arkansas. These examples of geographically uneven, often cross-cutting race-place associations suggest that urban-rural and regional dichotomies used in national-level child mortality studies can mask high-mortality environments (Dribe et al. 2020).

Early twentieth-century child mortality differentials further reflected [parent] socioeconomic status (SES), variously measured as occupation, income, and education. Child mortality, inversely associated with higher occupational rank/occupational income (Elman et al. 2019; Dribe et al. 2020; Haines 1985; Karbeah and Hacker 2023; Logan and Parman 2018), was highest among agricultural laborers, an occupation with “disproportionate representation … of blacks and foreign-born whites” (Preston and Haines 1991: 119; also, Haines 1985). But while Preston and Haines (1991) could calculate child mortality indexes for rural White women in all spousal professional, technical, managerial/official, clerical, and sales occupation categories, they combined categories for rural Black women because too few spouses occupied them. High occupational rank, especially among southern Black Americans, was historically present but rare (Anderson 1988). Regarding education – 1900 and 1910 Censuses report literacy – White mothers’ literacy (and spouses’, if measured) was protective against child death (Dribe et al. 2020; Elman et al. 2019; Karbeah and Hacker 2023; Logan and Parman 2018). Black mothers’ literacy could be protective (Elman et al. 2019; Logan and Parman 2018), although studies report mixed findings (Preston and Haines 1991).

However, recent work questions human capital comparisons by race-related categories in the context of the Jim Crow South, as human capital was (co) determined by race (Walsemann et al. 2022). A more robust stratification measure is wealth (Darity and Mullen 2020; Karbeah and Hacker 2023) although comparability again matters. Southern Black American farm (property) ownership was essentially null at Civil War’s end, reached an approximate 20 percent peak around 1910 and then rapidly fell (Higgs 1977). Southern Black American wealth in the era of Jim Crow, like high occupational rank, was present but rare due to legally structured racial inequities (Darity and Mullen 2020).

While adjusting for socioeconomic factors alongside place-related factors narrows the early twentieth-century Black-White child mortality gap, much remains unexplained (Preston and Haines 1991). Studies further consider structural determinants, including living standards, public health, and medical knowledge (Beardsley 1987; Preston and Haines 1991). However, the *contemporary* race-related gap in infant/child mortality remains unexplained – net of careful accounting of maternal and child factors – despite more-precise modeling of living standards (e.g., housing quality, nutrition), health utilization (e.g., prenatal care), and maternal stress-induced factors (Owens-Young and Bell 2020). Consequently, studies increasingly examine racism – a structural determinant of child mortality – because it is embedded in the institutions that allocate living standards, healthcare access, and life stressors (Owens-Young and Bell 2020; Phelan and Link 2015; Williams and Mohammed 2013).

An important indicator of structural racism in historical child mortality research is residential segregation, although few studies incorporate rural places. Logan and Parman (2018) report segregation widened the race-related child mortality gap in urban and rural settings (1900,1910 data), conditional on population composition measured as *county proportion Black*. Their segregation measure, alone, did not reach statistical significance. Karbeah and Hacker (2023) report both segregation and population composition (*Enumeration District proportion Black*) widened the child mortality gap in urban and rural settings, net of each other and combined. Despite somewhat inconsistent findings, results in both studies suggest a notable association between population composition and child mortality. As noted by Massey et al. (2009: 75; 1995), US segregation prior to the mid-twentieth century primarily manifested as macro-segregation at state and county levels. For example, macro-segregation in 1860 – indicated by the US *county proportion of Black persons enslaved* – remained highly correlated with US *county proportion Black* in 1930 and 1970, at *r* = 0.88 and 0.82, respectively (Kramer et al. 2017: 612). This persistent state-county population composition correlation, from the mid-nineteenth through the mid-twentieth century, is important for our study: macro-segregation in the American South, before and after the Civil War, geographically overlapped southern plantation agriculture (Aiken 1998; Beckert 2016; Higgs 1977: 24–26). As Census officials noted (U.S. Census Bureau 1916: 16), “In the great majority of the counties for which plantation statistics are presented the Negroes constituted at least half of the total population.”

Bioarcheological and historical–epidemiological small-area studies of the American South point to correlates of child survival, including on plantations. Most report variable child mortality levels within-race/ethnicity and within-place: different mixes of race/ethnic, household, and kinship practices, in addition to characteristics of localities, combined to shape child exposure and vulnerability to poor health and mortality (Brown 1979; Gutman and Fliess 1996). Rural Black children’s mortality risk might equal or exceed that of urban peers where plantation exposure occurred (Franklin and Wilson 2020; Thompson 2017). These studies also stress the importance of investigating biophysiological processes relevant to the geographic South, including the following: (1) chronic diseases of malaria, hookworm, and pellagra (Clay et al. 2019; Humphreys 2009); (2) syndemic, synergistic disease co-occurrence (comorbidities), including (mal)nutrition; and (3) divergent disease-age progressions to mortality, across diseases, among children and youth (Beardsley 1987; Brown 1979; Franklin and Wilson 2020). Finally, most report rising mortality among southern Black children and adults after 1880, coincident with poor and deteriorating conditions, including Jim Crow-era racialization (Beardsley 1987; Downs 2012; Menzel 2024; Smith 1995). Our study focuses on these conditions.

## Historical background

### Postwar (Re)institutionalization of Plantation Agriculture.

The Jim Crow regime carried forward, to the mid-twentieth century, racial ideologies and economic projects rooted in the past regime of Enslavement (Omi and Winant 2015). Most of the US Black population, prior to the Civil War, lived in southern counties where rich soils supported large-scale commercial farming; middling to large plantation owners (hereafter planters) had enslaved them to produce cotton, tobacco, rice, and other exports (Johnson 2013; Omi and Winant 2015). Planters, as political elites, retained a strong grip on the means of plantation production through laws and customs regulating land ownership, banking/factorage financing, and the labor system of Enslavement itself; all laws tilted in their favor (James 1988; Johnson 2013). The lost Civil War weakened their hold on power but, by Reconstruction’s end (1877), a business-oriented planter class (re)gained state-local sovereignty across southern states (James 1988; Woodman 2001).

Prior to the Civil War, most of the southern Black population, enslaved, lived in plantation counties (Higgs 1977; U.S. Census Bureau 1916), including in Arkansas (Hanson and Moneyhon 1989). Emancipation created a “labor problem,” perceived by the planter class as the loss of a Black labor supply though, as Woodman (1979) noted, Enslavement’s demise involved a *loss of the coercive social organization* of laborers, not a loss of laborers. From the perspective of the formerly enslaved, the problem involved labor autonomy rights amidst federal retrenchment of promised land grants of forty acres (Darity and Mullen 2020). State legislatures across the South – responding to planters’ demands for labor, freedmen’s resistance to working under Enslavement-style labor practices, and states’ catastrophic postwar economic losses—incrementally passed laws, from the 1870s to 1900s, to support contract-based household labor systems and to increase plantation productivity to levels formerly obtained through Enslavement (Roback 1984; Woodman 1979).

The rising prevalence of contract-based tenancy occurred in concert with a reorganization of plantation-mode farming. Pre-Civil War plantations were single, large farms; they comprised about one-third of southern land devoted to farm production (Ruef 2004). By 1910, southern plantations held about half of US farms as subdivided farmlands or “multi-farm” enterprises (U.S. Census Bureau 1916). Each plantation held five or more seasonally-contracted tenant households on one tract of land, overseen by one owner/manager (Brannen 1924). Seasonal contracts dictated the crops that households could plant, household members’ work obligations, and remuneration, including housing/garden rights and food credits (Jones 2010; Woodman 1979).

Moreover, the southern Plantation Belt, itself, dynamically expanded southwest after the Civil War (Brannen 1924). Expansion partly reflected the rising entrepreneurial orientation of a new planter class that partnered with merchants, elected elites, and corporations headquartered inside and outside of the South (Ruef 2004; Woodman 2001). The expansion also reflected a southwesterly migration flow of rural laborers – including approximately one-eighth of the post-Civil War Black population – from the South Atlantic to Trans-Mississippi states (Matkin-Rawn 2014; Woodson 1918/2002). Most of these postwar southern Black migrants (to 1910) settled in Arkansas (Gordon 1993; Matkin-Rawn 2014), primarily in Mississippi Delta plantation-holding counties.

### Geographic-Ecologic Disease Environments.

Most southern states contain distinct geographic-ecological subregions (McKenzie 1994; Wright 2006). A Belt of rich-soiled, high-cost farmland, devoted to large-scale plantation production, cut across southern states in 1910 (Aiken 1998). This Plantation Belt extended from Virginia to Texas, holding nearly 400,000 predominantly Black tenant farm households (US Census Bureau 1916). Land surrounding this Belt contained poor-quality soils that could not support large-scale farming: mountainous Ozark/Appalachian (Upcountry) and pine-forested Coastal Plain counties (McKenzie 1994). Most southern White populations, concentrated in these counties prior to and after the Civil War, could not afford to purchase higher-quality, higher-priced plantation farmland (Blevins 2002; Bolton 1994).

**Figure 1.**
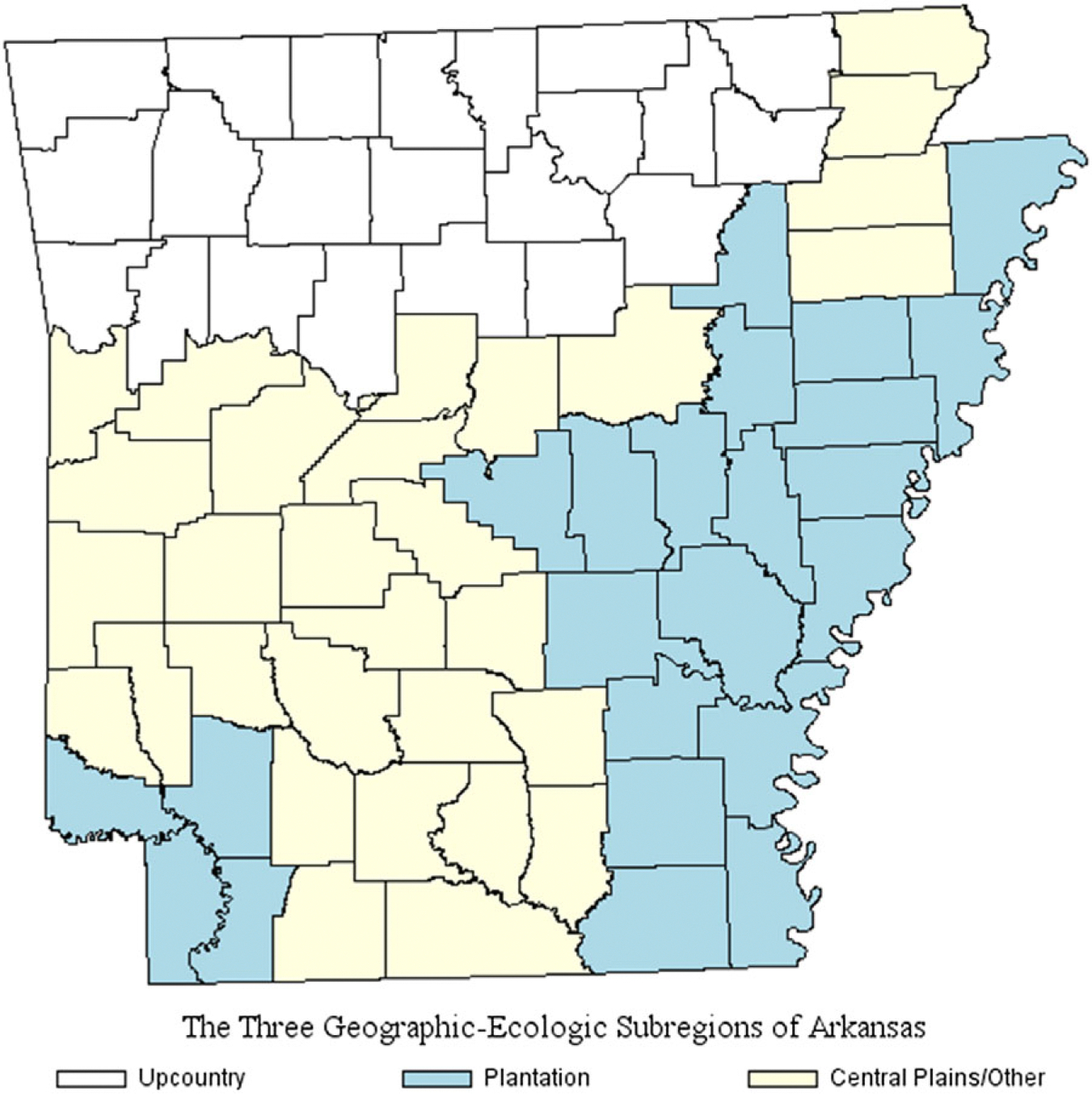
Three geographic-ecologic subregions of Arkansas, 1910. *Sources:* Plantation counties, Mandle (1992); Upcountry counties, Blevins (2002: 5). National Historical Geographic Information System (Manson et al. 2019).

Fig. 1 maps subregions in Arkansas (1910). Most postwar Black migrants settled in the Mississippi Delta and other (low-lying) plantation-holding counties (Elman et al. 2020; Gordon 1993; Matkin-Rawn 2014). White populations primarily inhabited Upcountry counties (Ozark, Ouachita ranges) (Blevins 2002); remaining inhabitants lived in Coastal Plain (pine-forested) and mostly undeveloped counties (Moneyhon 1997; Whayne 1996). Upcountry and Coastal Plain residents hunted, fished, and grew food crops for home use (subsistence) although many, by 1910, had shifted from subsistence to small-scale commercial farming (Moneyhon 1997; Woodman 2001).

These different subregional ecologies (Fig. 1) supported different childhood disease environments. Prior to the Civil War, planters and enslaved alike feared that a move “down the river” to Mississippi Delta riverbeds could be a death sentence, due to malarial disease (Johnson 2013; McMillan 1990). Postwar migration to Arkansas, however, boomed due to opportunities to purchase low-cost rich-soiled farmland, find well-paying tenancies, start communities, and/or earn extra or off-season pay by clearing forests (Elman et al. 2020; Whayne 1996; Woodman 2001). Yet, postwar plantation development sustained Delta malarial endemicity well into the twentieth century: the hot, humid climate and standing water (ponds) produced by deforestation and levee construction for land development allowed *Anopheles* (mosquito carriers of malaria) to thrive (Humphreys 2001). The growing rural population density – clustered cabins on plantations and crowding within – supported malaria and other infectious disease transmission (Humphreys 2001; Maxcy 1923).

**Figure 2.**
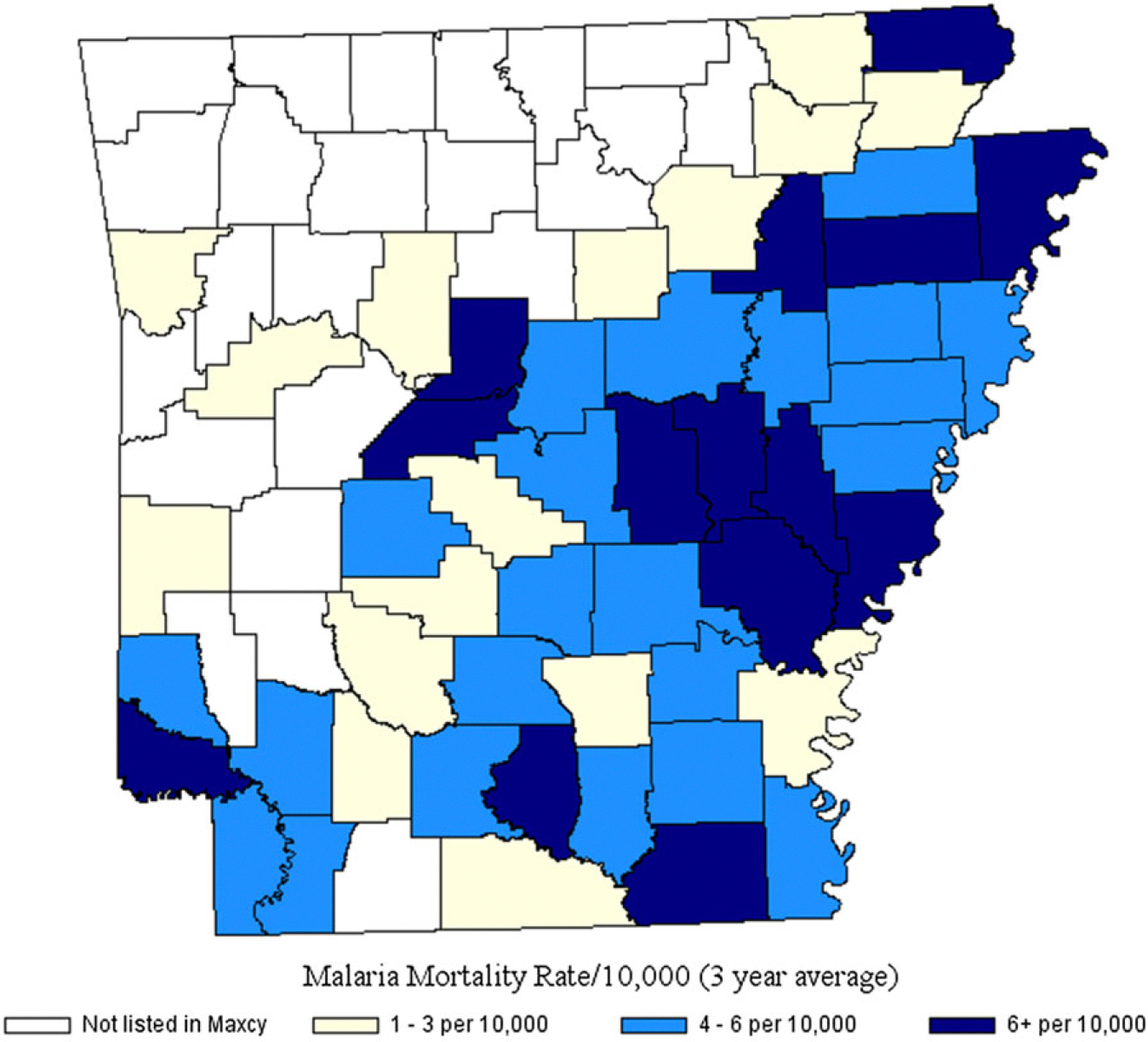
Malaria rates, Arkansas Counties (1919–1921). *Sources:* Maxcy (1923). National Historical Geographic Information System (Manson et al. 2019).

Malaria prevalence was highest in plantation-holding counties (Fig. 2). An Arkansas health survey (1913–1914) found one-third (38 percent) of cases in children under age 15 (von Ezdorf 1914). Endemic malaria, where present today, accounts for about 25 percent of age five-and-under all-cause mortality (Sachs and Malany 2002); child survival is especially uncertain in harsh environments (Whitty et al. 2005). Tuberculosis (TB) prevalence also was high on plantations due to rural density, proximity to animals and, less commonly, exposure to contaminated milk (Thompson 2017). While TB is associated with urbanicity, primarily-rural Arkansas in 1910 had among the highest TB mortality rates in the nation with most succumbing aged 5–30, female, and Black (Floyd and Bates 2023). Bioarcheological evidence from plantation cemeteries reveals that Black children’s poor nutritional status reduced their resistance to high-fatality malaria, TB, and other diseases (Franklin and Wilson 2020; Rose 1989; also, Woods 2009).

Most White Arkansans, in contrast, lived in Upcountry and Coastal Plain/Mixed counties where the cooler, dryer climate, higher land elevations, and sparser rural populations reduced infectious disease transmission (McMillan 1990: 69). Wealthy White Arkansas migrants, prior to and after the Civil war, *selectively* settled on poorer-quality land, far from plantation production, *to avoid* exposure to malaria and other diseases. Sandy Upcountry and especially Central Plains ecologies, however, were breeding grounds for hookworm, ascaris, and other soil-borne parasites (Franklin and Wilson 2020). Hookworm prevalence was highest among southern school-aged children (Elman et al. 2014); while less lethal, the infections impaired health and human development.

Important for this study, Black Arkansans primarily lived in plantation-holding, malarious Delta counties while White Arkansans primarily lived in subregions shielded from high-fatality infectious disease transmission. Subregional ecologies’ different productive modes also differently shaped socioeconomic mobility among children, by race. We turn to this below.

### Socioeconomic Position and the Agricultural Ladder.

Half of southern White and about 20 percent of Black farmers owned farms in 1910; about 40 percent of White and 75 percent of Black farmers were tenants (Higgs 1982; also, Tolnay 1984). However, higher proportions of Black than White tenant farmers were plantation tenants (U.S Census Bureau 1916). While child mortality studies examine stratification using measures of property ownership and wealth, other literature finds that agrarian socioeconomic stratification was dynamic, resembling an Agricultural Ladder. Position on the Agricultural Ladder was highly intercorrelated with income, wealth, occupational rank, and socioeconomic mobility (Alston and Kauffman 1997; Alston and Ferrie 2005).

Farm owners on the top rung were wealthier (owned livestock and tools) and had more control of crop mix (what to grow) and household members’ roles, including schooling for children (Guest 1981). Cash tenants, the next lower rung, paid for land use and owned livestock and tools; their relative autonomy regarding crop mix and children’s roles was more contingent on external conditions (commodity prices) and household economic factors, including child labor demand (Guest 1981). Critically, share tenancy, the occupational category on the next lower rung, differed based on whether tenants were employed in plantation or non-plantation agricultural modes (Alson and Kauffman 1997; U.S. Census Bureau 1916; Woodman 2001: 807). The difference implicated occupational rank: by 1920, the 14th Decennial Census distinguished plantation share tenants – as low-rank sharecroppers, mostly Black – from higher-rank “true” share tenants, mostly White (Alston and Kauffman 1997; Woodman 2001). As Woodman (2001: 808) put it, Black and White farmers could be tenants and poor, but “class similarity was more apparent than real.” Black tenants were disproportionately wage-like laborers on business plantations; White small farmers, as “true” tenants, resembled agrarian small businessmen.

Most White share tenants, as true tenants, owned tools and livestock. Critically, most would build land wealth from farming and/or inheritance, thereby moving up the Agricultural Ladder to ownership, in a life-cycle pattern (Alston and Ferrie 2005, 1910–1940 data). In contrast, plantation share tenants, as sharecroppers, less often owned tools or livestock and were less remunerated for the skills that they brought to contracts (Brannen 1924; Woodman 1995). Their contracts left little choice about crop mix, home production of food, work hours, housing, medical care, and children’s education (Anderson 1988; Elman et al. 2020; Smith 1995; Tolnay 1984; Woodman 1995).

Black sharecroppers desired to move up the Agricultural Ladder, much as true tenants, but most remained sharecroppers for life (Mandle 1992; Woodman 2001). More confined to plantations than true tenants by aseries of Jim Crow laws (Roback 1984), Black farmers’ upward mobility on the Agricultural Ladder – as well as their ability to exit the Ladder by entering non-farm occupations – was limited. In Arkansas, early crop lien laws (1885) gave planters, bankers, and merchants the first slice of tenants’ crops to repay food purchases over seasonal contract periods. The laws produced economic precarity and debt among the poorest tenants, disproportionately Black, because the “first slice” often took the whole pie (McKenzie 1994). Convict leasing laws (1880–1890) limited primarily Black persons’ physical mobility to search for better jobs and created a new “unfree” labor class – Black convicts – of forced laborers in plantations, mills, and mines (Smith 1995). Arkansas contract enforcement laws (1907) more deeply tied tenants to seasonal contracts.

Exiting local plantation economies was also difficult for Black farmers. School segregation and the subjects taught limited their (and their children’s) occupational mobility into white-collar work (Anderson 1988). Upcountry towns’ drawing of color lines (adoption of sundown codes) limited geographic mobility by prohibiting entry and allowing violent removal of Black persons from White spaces, completely or by time of day. This ensemble of racialized economic and social policies at the twentieth century’s turn supported macro-segregation (residential concentration) of Black rural populations, in Arkansas and across the South, in counties with plantation-mode farming. We examine whether exposure to this agricultural mode and related conditions was associated with Black children’s mortality disadvantage. We expect to find that:

H1: The racial gap in child mortality was associated with plantation-style production and larger in Plantation subregion counties.

H2: The racial gap in child mortality was associated with health environments and larger in counties with higher levels of population density and malaria mortality.

H3: Child mortality was associated with socioeconomic position on the Agricultural Ladder and was higher among mothers living in tenant vs. owned farm households.

H4: Black mothers living in counties with higher Black farm ownership rates (e.g., collective resources) experienced lower rates of child loss.

H5. Black mothers experienced higher rates of child loss than White mothers due to less-measurable racial discrimination, net of positionality in productive mode, economic rank, and proximate determinants, such as human capital (e.g., literacy).

## Data and methods

We adopted a mixed-methods approach and collected and analyzed qualitative and quantitative data sets separately (Creswell et al. 2017). Prior studies informed the statistically testable hypotheses in the quantitative portion of this study and the guiding concepts in the qualitative portion of this study. The quantitative portion estimates the Black-White child mortality gap in 1910 Arkansas, based on Census-measurable conditions related to child deaths. The qualitative portion cannot speak directly to the causes of relative differences in Black-White child mortality but can provide insight (as emergent themes) about the life conditions under which Black children faced high risks of death. We consider both the quantitative and qualitative portions of our study to be exploratory. Our goal in adopting a mixed-methods approach is to produce a richer set of findings about Black children’s mortality risk than obtainable from Census data sources or oral accounts, alone.

### Quantitative Data.

We drew an analytic sample of ever-married, parous (had borne at least one living child) Black and White Arkansas women ages 15–49 (*n* = 234,811) from the 1910 Complete-Count Integrated Public Use Microdata Series (IPUMS) (Flood et al. 2015). Census enumerators only queried ever-married women about childbearing histories; non-parous women were not at risk for child death(s). We used Federal Information Processing Standard (FIPS) Arkansas county codes of place of residence at enumeration to link the IPUMS sample to Arkansas county-level data (*n* = 75 counties) taken from the 12th (1900) and 13th (1910) Decennial Censuses (Haines and ICPSR 2010), and from other data sources (listed below). We obtained Arkansas plantation counties from Mandle (1992; see Online Supplement for further description). We obtained Upcountry counties from Blevins (2002: 5). We obtained County malaria mortality rates, as moving averages, calculated by Maxcy (1923). We used the *IPUMS National Historical Geographic Information System: Version 13.0* (Manson et al. 2019) for map construction.

Dependent Variable and Method. The 1900, 1910 US Censuses reported ever-married women’s number of children born and surviving. Most child mortality studies using these Censuses employ indirect techniques to estimate child mortality indexes as ratios of observed child deaths among currently or first-married women for given marital duration groups, compared to expected deaths in these groups as derived from life tables (Preston and Haines 1991). A few studies have used Census 1900, 1910 IPUMS microdata directly, differencing numbers of children born and surviving to estimate number(s) lost (Logan and Parman 2018) or proportions lost (Elman et al. 2019; Guinnane et al. 2002; Hacker et al. 2023).

Most indirect-method analyses restrict samples to currently married women because separated/widowed/divorced women did not report marital durations. They may also restrict samples to first-married women because remarried women report current marital duration but have children born/surviving from all unions. However, we did not wish to exclude not currently married or remarried parous women from our sample: they were more likely to be Black (Preston et al. 1992) at a time when Black women had a higher risk of child loss (Preston and Haines 1991). Additionally, we did not want to selectively eliminate not currently married women because, in our sample’s agrarian world, currently married women’s households commanded the most land wealth and/or better tenancy terms. Finally, we wanted to consider each woman’s number of child deaths in context of their parity (number of births). Southern Black farm women had higher fertility rates than White peers – their total fertility rate in 1905–1910 was 7.4 (Tolnay 1987); higher parity/longer childbearing spans may have elevated their risk of child loss.

Given these issues, we used count models to examine a dependent variable, *number of child deaths,* constructed as the difference between number of children born and number surviving. We used the number of children born, logged, as an exposure or offset variable to examine mothers’ rates, and race-related differences in mothers’ rates, of child loss. We interpret our regression findings as own-mother child mortality rates (Zwilling 2013).

### Women’s characteristics.

The 1910 Census reports separate categories of Black and Mulatto women; we coded both as *Black* with White as the reference category. Women’s biologic reproductive health differs by age; we adjusted for *age* in models. We modeled women’s education and migration status as *literacy* (ability to read and write) and *birth in another state,* respectively. Marital status includes *currently married* with separated/divorced/widowed as the reference category.

### Household-level variables.

The 1910 IPUMS reports ownership of dwellings and whether dwellings are farms but does not distinguish tenant farm type (e.g., cash or share). We formed categorical variables *tenant farm* (not owned) and *non-farm* (owned or non-owned), with farm ownership, the top rung of the Agricultural Ladder, as the reference. We adjust for *rural* residence (household-level); urban household location is the reference.

### County-level variables.

**Figure 3.**
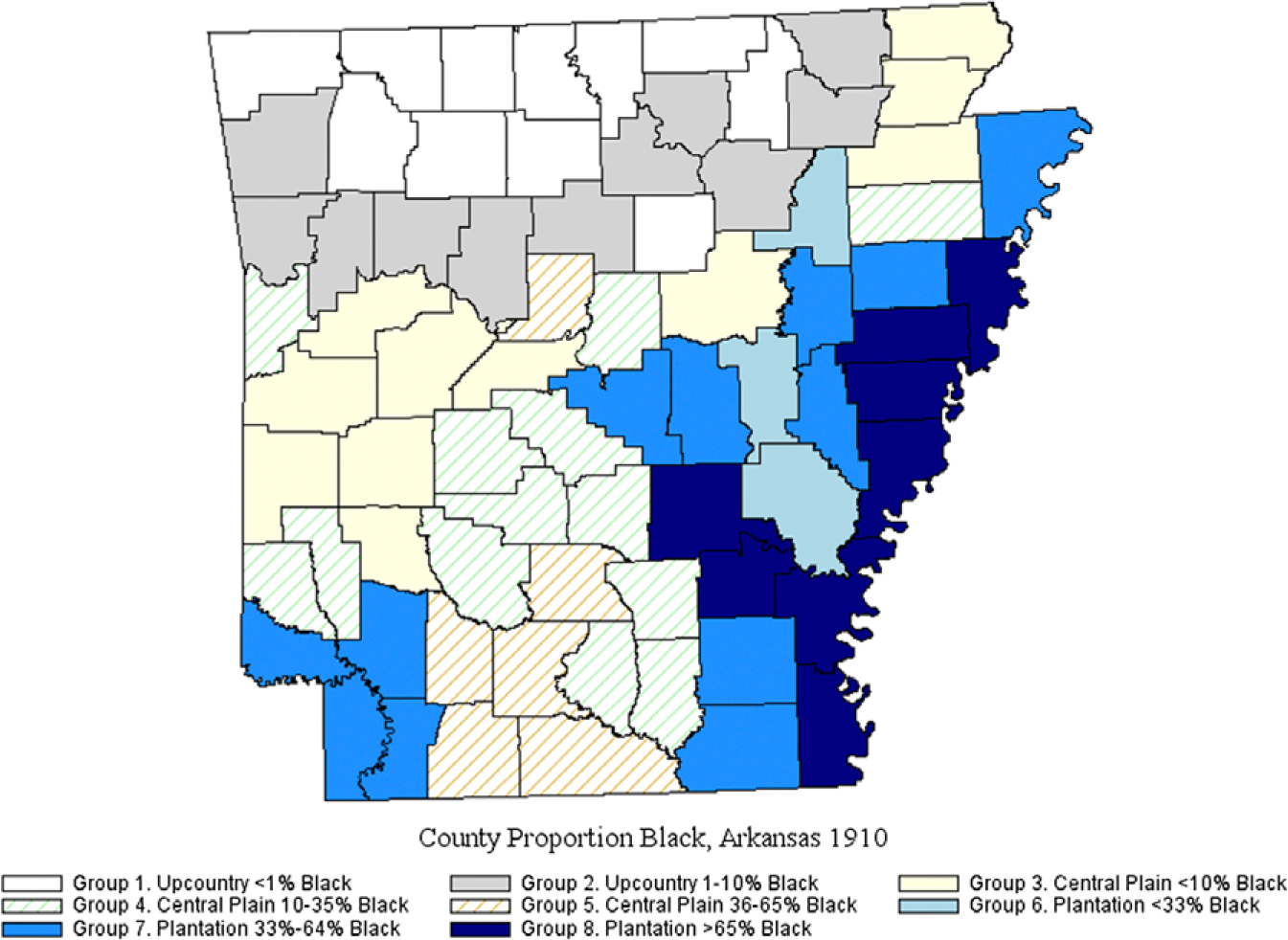
Arkansas County percent Black by subregion, 1910. *Sources:* Plantation counties, Mandle (1992); Upcountry counties, Blevins (2002: 5); Haines and ICPSR (2010); National Historical Geographic Information System (Manson et al. 2019).

We draw 1900 and 1910 county-level measures from Haines and ICPSR (2010). We wished to further differentiate segregation levels across Arkansas Subregions. We examined, using county-level data only, 1910 *county proportion Black* univariate distributions for each Subregion and then formed dichotomous Group 1–8 variables, based on data cut-points in each Subregion. For example, the White populations were concentrated in Upcountry counties. As the *proportion Black* did not exceed 10 percent in any county, we used <1 percent and 1–10 percent cutpoints form dichotomous indicators of Upcountry Groups 1–2. For *proportion Black* in Plantation subregion counties, we used <33 percent, 33–64 percent, and 65 percent+ cutpoints to form dichotomous indicators of Group 6–8. Fig. 3 illustrates the cross-classification of Subregion by population composition.

Other variables include 1910 *county population density (logged)*, 1910 *county proportion of farms that were tenant farms,* and a 1900 Census lagged measure, *proportion of county farms that are Black-owned farms. Malaria mortality rates* as calculated by Maxcy (1923 also, Elman et al. 2019) are 3–year averages, 1919–1921, for counties with rates greater than 1/10,000.

### Statistical analysis.

A count-dependent variable is best estimated by Poisson regression, a method that assumes regression error distribution is not over-dispersed (that the conditional mean equals the conditional variance). Preliminary analysis found the assumption violated; a negative binomial (NB) model, which relaxes the distributional assumption, best-fit the data. We estimated three statistical models. The first adjusted child mortality rates by race. The second model added individual-level, household-level, and geographic Group dichotomous variables. The third model examined model 2 variables but substituted county-level for Group dichotomous variables, as using both introduced collinearity. It is possible to analyze the third model, with county-level data, using Random Effects maximum likelihood-based methods that generate subject-specific findings, or with Generalized Estimating Equation (GEE) quasi-likelihood methods that generate population-averaged findings (Hubbard et al. 2010). Our analysis is exploratory; we used GEE estimation, consistent with our aim of assessing a population-level race difference in Arkansas child mortality. We found nonlinearity in equations due to race interaction terms and hence calculated predictive margins to facilitate the interpretation of race coefficients. We used SAS 9.4 Proc Genmod and the SAS Margins Macro. All model findings are based on cluster-robust sandwich standard errors (counties) and, due to the large sample size, *p*<0.01 (two-tailed) statistical tests.

#### Qualitative Data.

We used qualitative materials from two sources: (1) Federal Writers’ Project (FWP) interviews conducted in the 1930s with formerly enslaved women and, (2) Behind the Veil (BTV) interviews conducted in the mid-1990s with elderly Black women and men who grew up in the Jim Crow South. These sources provide insight into Black children’s lives in Jim Crow-era Arkansas. An Online Supplement provides a interviewee list.

We accessed the FWP-transcribed interviews from Arkansas in the Library of Congress Collection: *Born in Slavery: Slave Narratives from the Federal Writers*’ *Project, 1936 to 1938*. The total number of FWP Arkansas interviews was 696; we downloaded 317 narratives from women for analysis. Within these, we sampled purposively based on residency in early twentieth century Arkansas using a priori codes from quantitative variables as well as the research literature, including pregnancy/childbirth, childrearing, and references to health, work, and/or home/housing. This yielded a final sample of 80 narratives. We are sensitive to FWP reliability concerns about interviewer bias, recall bias, older age, and personal agendas of interviewees, but Arkansas accounts have less bias (Cantrell 2004)^4^ and are, as Simmons notes (2021: 313), “an important archival and evidentiary base for thinking through Black concepts of history, time, and generation.” We developed a codebook for the analysis using sampling codes and emergent themes discovered in the inductive process of open and axial coding of the text of each interview (Corbin and Strauss 1990). We used MaxQDA (versions 10, 11, 12, and 2020) software for data management and analysis.

The second source, Behind the Veil (BTV), consists of interviews conducted in the mid-1990s, held in the John Hope Franklin Collection of African and African-American Documentation, a Special Collection at Duke University’s Rubenstein Library. The BTV archives include 1,265 oral histories of older African Americans born in southern Jim Crow states (Chafe et al. 1990). We sampled BTV interviewees who were born or spent childhood in Arkansas around or after 1910; they provided recollections about themselves and their families of origin. We transcribed BTV data from 26 audio-recordings and/or Special Collection transcripts of interviews (if available); two authors reconciled differences between their transcriptions of materials. Three of the interviews included two interviewees, for a total of 29 individuals. The codebook developed for FWP analysis guided the coding process for BTV accounts, using NVivo (V.12) software.

## Results

Table 1 presents descriptive statistics for all mothers (Column 1) and Black and White mothers (Columns 2 and 3, respectively). Arkansas mothers bore on average, 4.11 children and lost one (1) child. Black mothers bore more (4.24 vs. 4.05) and lost more (1.36 vs. 0.88) children than White mothers. Compared to White mothers, Black mothers were less likely to be literate (71.2 percent vs. 92.8 percent) and currently married (78.3 percent vs. 91.1 percent). Almost half (42.3 percent) of Black and White mothers were migrants to the state. Most Black mothers (75 percent) lived in the Plantation Subregion; only 1.8 percent lived in the Upcountry. Black mothers were less likely to live in owned farm households (13.6 percent vs. 35.8 percent) and more likely to live in counties with denser populations – conducive to infectious disease spread – and higher malaria mortality.

### Child mortality: Statistical Findings.

Table 2 displays negative binomial count regression results, as child mortality incidence rate ratios (IRRs). The unadjusted IRR in Model 1 for Black mothers is 1.49, suggesting a 49 percent mortality rate increase among Black compared to White mothers. The predictive marginal race difference, indicating higher Black child mortality, is statistically significant (Footnote Table 2).

**Figure 4.**
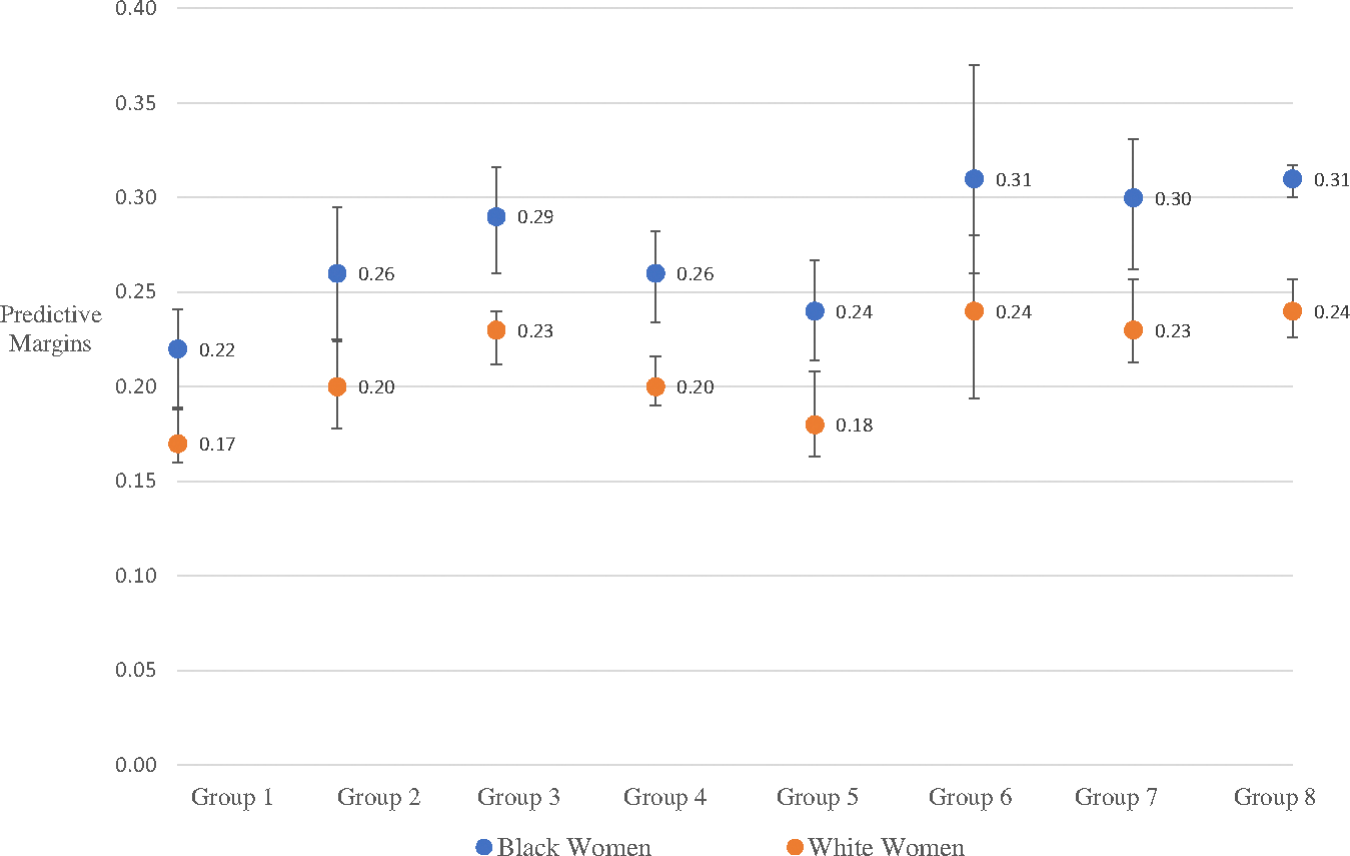
Predicted marginal effects of subregional groups on child mortality rates, by race.

Model 2 adjusts for women’s characteristics, household tenancy rank, and dichotomous geographic Subregional Groups. In Model 2, the IRR for Black mothers is 1.17, suggesting a 17 percent increased risk of child mortality net of other variables. Mothers in Plantation Subregion Groups 6 and 7 have the highest IRRs, but these IRRs are not statistically different from mothers in Group 8 (reference). In contrast, mothers in Upcountry Groups 1–2 have the lowest rates relative to Group 8.^5^ Fig. 4, based on Model 2, presents predicted margins of geographic Groups by race, showing child mortality highest among Black mothers, across Groups, but with a gap widest – differences ranging from .068 to .07 points higher – in Plantation Subregion Groups 6–8, respectively (*Hypothesis 1*).

In other findings, Arkansas migrants have higher IRRs, perhaps reflecting mothers’ reduced access to kin networks, community supports, or health impairments following Trans-Mississippi West moves under conditions of hunger, disease, and outdoor exposure. Living in a tenant versus farm-owning household is associated with higher child mortality (*Hypothesis 3*). Also, literacy statistically moderates the race gap. The predicted margins of literacy by race (not shown in the table) are associated with lower Black and White rates, but more so among White mothers: their rates are reduced by .044 points (–.044, .004 SE, *p*<.001) while Black mothers’ rates are reduced by .024 points (–.024, .003 S.E., *p*<.001), a statistically significant difference.^6^

In Model 3, substituting county-level associations, IRRs for women’s characteristics and household resources change little compared to Model 2. Net of this, child mortality is positively associated with county malaria mortality rates and population density (*Hypothesis 2*), the county proportion of tenant farms (*Hypothesis 3*), and is inversely associated with county Black farm ownership rates (*Hypothesis 4*). In examining average marginal effects, by race, malaria and county tenancy rates widen, while Black farm ownership rates reduce the child mortality gap.^7^ The race predictive margin remains statistically significant (footnote Table 2) (*Hypothesis 5*).

#### Qualitative Findings: Plantation Life and Health Environments.

We turn to qualitative data to explore Black children’s risks – especially in relation to plantation life in the context of the Jim Crow South – as voiced by older selves, mothers, and community witnesses. One theme that persisted among BTV participants exposed to plantation life was *field work.* Critically, all Arkansas Black and White farm households in 1910 – owned and tenant, across agricultural mode – used household members’ labor (Elman et al. 2020). Yet economic contributions varied by rank on the Agricultural Ladder. In non-plantation settings, and among farm-owning and higher-ranked tenant households, mothers had more discretion about outdoor work and its timing, and most children, less needed for fieldwork, attended school (Elman et al. 2020; Guest 1981). In contrast, most plantation tenants’ contracts required all members to work in fields, especially during the planting and harvesting months (Brannen 1924).

BTV participants recalled their own fieldwork when children. Le Ester Jones’ (BTV) story was common: “I was about seven, when I started working in the field. And before that I had to carry water to the people … being small that was my job, carrying food and water to the fieldworkers.” Mattie Bell Lowe (BTV), still a farmer when interviewed, also began field work at age seven: “I can remember the first time I started plowin’ … I was seven years old … Plowed all, I could plant the cotton right up on top of the row, I could use the cultivator, I could do all that … And me and my sisters would help [my mother] chop ‘til we’d catch up with Dad and plow the next day.”

BTV women also recalled difficulties, as mothers, in balancing fieldwork, child care, and housework demands. Taking infants/young children to fields limited their work productivity (hence remuneration) and their ability to supervise children. But leaving them at home in the care of older children (if any), reduced the number of household field workers and jeopardized child safety. In Hagood’s (1939) study of South Atlantic White tenant mothers, one mother reported losing two children, at separate times, while she picked cotton: each child, while minded by older siblings, walked, or crawled into the open fireplace.

Other themes, of *(poor) housing*, and *(lack of) food* illuminate plantation disease/health environments and their potential association with child mortality/survival (*Hypothesis 2*). Plantation owners/managers assigned housing by tenancy rank – and by color. Low-ranked tenants’ housing lacked indoor plumbing, sanitary (closed system) outhouses, window screens, even solid walls, roofs, and floors (Humphreys 2001; Jones 2010; Smith 1995). Katie Swanigan (BTV) lived in a “little ole three-room house … Outdoors toilets. Pumps, where you pump your water … Jus’ common houses, there’s board houses – most of ‘end was raggly … We used to could feed the chickens through the floor.” Such housing, combined with field work, left plantation mothers and children – at work and at home – highly exposed to malarial mosquitos and the diseases associated with poor sanitation (Humphreys 2001).

Regarding food, children in southern Black and White farm households, owned and tenant across agricultural modes, risked malnutrition, especially if their households practiced monocrop production (growing crops for market, not household, use) (Clay et al. 2019; Fite 1984). But farm owners and high-tenancy rank households, Black and White, owned livestock that produced eggs, milk, and meat; they might more easily forgo growing for home use. Willie Clyde Tims (BTV) lived on his grandparents’ owned farm in Coastal Plain Columbia County where they grew “cotton, corn, peas, and potatoes, [and] fruits of all kinds … and vegetables also for marketing, for selling.” Samella White (BTV) grew up in the Plantation Subregion and recalled, “It was plantations out there but I happened to be fortunate enough to live on my father’s, on a little bitty farm … And uh, we lived in a … little, little white house. My daddy he used to raise, all kinda, just animals you would see. Hogs, cows, chickens, ducks, geese.”

Subregional ecologies also mattered. Open range and forest surrounded most Upcountry and Coastal Plain farms, owned and tenant; roaming wild livestock provided households with free access to protein from meat and dairy (Moneyhon 1997). Plantation tenants had less open-range access to food and some plantation contracts forbade tenants from keeping gardens and livestock. Georgia Ford (BTV) recalled, “Back in our days, some white folks say [we] can have a garden just no chickens … Come Christmas time in hole, couldn’t get no food. Eight people on a farm made good cotton that year, went to the store and was told [they were] in debt, couldn’t get nothing.”

Plantation contracts requiring purchase of food, on credit, from plantation stores run by planters and/or merchants further limited plantation children’s food intake (Brannen 1924; Smith 1995). Over 90 percent of southern Black and White plantation tenants in 1910 used these commissaries, although White tenants had better legal standing to contest planter/merchant terms (Brannen 1924). The high-cost, low-quality food in commissaries, in conjunction with mothers’ reduced time for meal preparation due to fieldwork and contract-required monocrop production, resulted in “sharecropping families [subsisting] on a substandard diet of meal, molasses, and fatty meat, especially in the winter and spring” (Jones 2010: 85).

Plantation subsistence left tenants malnourished during the growing season (Dirks and Doran 2001). Many then faced winter starvation if contract-settling at fall harvest with planters/merchants did not leave enough to purchase winter food (Dirks and Doran 2001). BTV participants report that, as children, they were often hungry. Their parents limited food purchases, fearing indebtedness to planters through commissary use (also Jones 2010: 80). Mothers under these circumstances might prioritize their own nutritional needs last, choosing to feed husbands and children before themselves. Annie Floyd (BTV) noted, “We had problems, sometimes we didn’t have enough food on the table. I would fix it … put it on the table and walk out until my husband and the children eat. If there was anything left then I eat. If not, I did without until maybe I could scrap together something for the next meal.”

Mothers also took in washing/ironing and clothes-making or took outside domestic jobs – in addition to performing fieldwork – to help feed families. Dora Strong Dennis (BTV) recalled, “they called it lay by the crops, Mama would start to wash and iron so we could eat, so she could feed us, because they’d cut your groceries out, because every two weeks Mama would go to the commissary, they called it, and get our weekly ration, they said, and sometimes it would last and sometimes it didn’t.”

White tenant farm women with limited household resources might also increase workloads by taking in sewing or selling garden and dairy products. But sales were mostly household surpluses (Hagood 1939), and they rarely took in washing, an arduous task with racialized status; even the poorest rural White households sent laundry to Black laundresses (Jones 1988).

Recalling Racialized Institutions: Tenancy, School, Family Formation. Jim Crow-era policies carried forward pre-Civil War barriers to Black human capital accumulation involving education (Anderson 1988; Jung 2020). The oldest FWP mothers in our sample, born around the time of Emancipation were prevented, by law or war disruption, from learning to read and write. There were exceptions, as Laura Thornton (FWP) explained, “ I never learnt to read and write. In slave time, they didn’t let you have no books. My brother though was a good reader. He could write as well as any of them because he would be with the white children and they would show him.”

Black parents during Reconstruction, in Arkansas and across the South, eagerly established schools for children although White persons might burn Black schoolhouses and/or persecute teachers. Rose Adway (FWP) recalled, “After Mr. Lincoln [set] da slaves free, dey had Northern teachers down South and they were called spies, and all left the country.” In the Arkansas Plantation Subregion, Black parents supported education. Their children’s school schedules, however, followed planters’ field labor demands (Jones 1988). Elizabeth Tunage (BTV), born in the Plantation subregion town of Fargo, recalled

Well, we went to school around six months in the winter. Now that was the only thing that was kind of aggravating and I couldn’t understand. The white kids would start to school in September and go ‘til May. We didn’t start school until sometime about the last of October or about the middle of November somewhere along in there, and we went until it was time to start in the fields in the spring. And then we would go to the fields.

Cleaster Mitchell (BTV) noted that plantation owners “would come tell your parents, ‘You go down and get the kids. I got something for them to do.’ That was it. You went.”

Our statistical models find literacy less protective against mortality for Black children than for White children (Table 2). BTV participants recalled being pulled from school to field work; this suggests that Black mothers’ weaker literacy-child mortality association may partly reflect their own earlier life course “trade-off” involving school versus work. Put differently, the benefit Black mothers might have gained from their own education, including improvement in their children’s health, was likely reduced by their greater exposure to unhealthy field work when young children. Such a maternal trade-off, to the degree that it occurred, was a structural determinant of Black children’s health precarity: rooted in a racialized tenancy system that disproportionately tied Black children to field work more than education (Anderson 1988; Jones 2010; Jung 2020). Thus, the quantity and quality of education was lower for Black than for White southern Americans, among both adults and children.

Moreover, southern planters preferred contracts with male-headed tenant households with children; Black youth with little capital accumulation and unequal educational opportunities married and formed families earlier than White youth to enter low-ranked farm tenancies (Landale and Tolnay 1991). As low-ranked tenants, especially on plantations, they faced further barriers to capital accumulation (Darity and Mullen 2020); most did not reach ownership on the Agricultural Ladder, in life-cycle pattern (Alston and Ferrie 2005). The ensemble of Jim Crow-era racialized laws and discriminatory practices, intertwined across institutions, worked to reduce maternal resources, such as human capital, which might mediate child survival.

Community supports, child socialization to exclusion. Statistical analysis reveals an inverse association between county prevalence of Black farm ownership (collective wealth) and Black mothers’ rates of child loss (Hypothesis 4), suggesting Black communities with greater resources collectively helped one another. Oral accounts report that, in Arkansas Black communities, individuals, and families, without hesitation, reached out with offers of support to others in need (Elman et al. 2020). Geraldine Davidson (BTV) described: “The black community was more or less knitted. [Whatever was needed] the community came together and would help out, and I thought that was great growing up in that time.” Annie Floyd (BTV) recalled, “Doctors were scarce, money scarcer … Mama would sit with others [who were] sick, stay all night. … in the community, we shared … This was the only way to survive. Send kids to borrow, bring food. We would find out about others, no phones then, so and so is sick we found out by way of sharing food and visiting.” Oliver Williams (BTV) explained that “people worked together and they looked out for one another. Your welfare was mine, mine was yours. That’s the way they lived.”

Black community support, however, could not outweigh racialized structural barriers and practices of the Jim Crow South. Despite adjustments in statistical equations, Black children’s survival disadvantage remained (Hypothesis 5). BTV participants recalled many racialized conditions with harmful effects on child health; due to space constraints we highlight two. The first condition was a lack of health care. While medical care prior to 1910 was not a major factor in the US health transition, MDs gained considerable knowledge during the Civil War and the postwar period (Downs 2012). In 1910, children (and adults) benefitted from surgical techniques and medical treatment of burns, gashes, and broken bones. Southern MDs also performed the public health tasks of inoculation (for smallpox), disease surveillance, quarantine, and they distributed quinine for malaria (Elman et al. 2024). Lack of medical care was an emergent theme in BTV accounts. While White mothers talked about medical debt as a burden (Hagood 1939), BTV participants recalled a lack of any care (Simmons 2021). Le Ester Jones (BTV) recalled, “There was a lot of sharecroppers. When a member of the family got sick, it wasn’t no money for no doctors. … They stayed up with their sick and cared for them their selves. Lost small children and babies, the heads of the house, ‘cause they wasn’t able to have a doctor and they just had to depend on each other.”

A second condition was Black (White) children’s socialization to low (high) racialized status to sustain White supremacy (Jones 1988). Cleaster Mitchell (BTV) described the key interactional rule: “have no emotion at all because even just your expression sometimes always cause you a lot of trouble. You couldn’t react to anything. It was bad.” Willie Clyde Tims (BTV) explained how Black children learned this system: “You kind of gained that from the teaching as you grew older and as you learned the language of signs and expression …of being careful in how you would express yourself and what you would say and how you would act … you would not act in a way that would not make white persons feel that you didn’t know they were white.”

These racialized etiquettes persisted in adulthood. Delores Twillie Woods (BTV) noted

You didn’t get too old for them to call you a boy and girl or uncle and aunt … A lot of times you couldn’t say nothing, but it always made you made mad inside. If you couldn’t say it on the outside. You know. You didn’t want nobody saying uncle and aunt to you when you ain’t no kin to ‘em.

We know, today, that these discriminatory practices constitute stressors that negatively impact pregnancy and child survival outcomes, including probabilities of high-risk, low-birth-weight infants (Williams and Mohammed 2013).

While Black communities could not outweigh forces producing children’s deaths, they could memorialize lost children, regardless of age or stage at birth or death. A haunting litany of lost children repeats throughout the FWP and BTV accounts as interviewees recounted the numbers of children birthed and the much lower numbers still alive. Whether women had one child or many, even those lost through miscarriage and stillbirth might be included in the recounting. As Simmons (2021: 326–327) observed in her analysis of generationality and Black relationality in FWP oral histories:

Infant death also comes to the foreground when women identify the number of children they had or when they enumerate their mother’s pregnancies … [a] tendency for women to include babies who had passed away among their children signals the ways in which the women remembered the dead as well as honored their bodily experience in pregnancy and childbirth.

Sarah Anderson (FWP) simply noted that “All my chillum dead cept one son. He was a twin.” Some gave counts of their siblings, living and dead. William Thomas Malone (BTV) stated, “I had so many brothers and sisters … they died before I was born or when I was a baby and I don’t remember them … eighteen brothers and sisters. I don’t have a brother nor sister living.”

Bioarcheological study of the excavated plantation community cemetery in Cedar Grove, Arkansas, found about half of Black children died after age 5, the age traditionally measured in childhood mortality research (Franklin and Wilson 2020; Rose 1989). Many of their burial sites were, at the time, being planted over by landlords or otherwise hidden, but infant and child deaths were a shared part of life. Black communities created special songs and burial rituals for children who were gone. Mary Jane Drucilla Davis (FWP) could recite, at her end of life, the first song she remembered from when she was three years of age:

Oh, it was so mournful. And let me tell you what they’d do. They’d all march one behind the other and somebody would carry the baby’s casket on their shoulder and sing that song:

‘Little baby’s gone to heaven

To try on his robe

Oh, Lord, I’m most done toiling here

Little baby, m-m-m-m-m-m.’

## Discussion

We examined Black children’s survival disadvantage in Arkansas at the twentieth century’s turn. Arkansas was a rural, agricultural state; however, the state’s geographic subregions contained different geographic–ecologic productive modes and disease environments. State laws passed after Reconstruction – to buttress plantation productivity with tenant labor and to segregate work, travel, schooling – had (re)installed racialized economic dependency under a rising “American-style apartheid” (Jones 2010: 100). The state’s Black population primarily lived in high-productivity plantation counties; most of the state’s White population lived in counties where farm ownership and true tenancy rank prevailed (Woodman 2001). Using a mixed-method design, we examined Black-White child mortality rate differentials and used oral accounts to explore Black children’s life conditions under plantation agriculture and Jim Crow-era practices.

Our first finding of Black (and White) racial concentration in Arkansas (Fig. 3), is consistent with a US pattern of macro-segregation prior to 1960 (Massey and Hajnal 1995, 2009). Our second finding, that Black-White child mortality differentials were widest in the Plantation Subregion counties holding the state’s largest Black proportions (Fig. 2, Groups 6–8, versus Groups 1–5) (*Hypothesis 1*), is consistent with studies that link population composition to Black-White child mortality gaps (Karbeah and Hacker 2023; Logan and Parman 2018). Additionally, Black women’s comparatively high rate of child losses across the Plantation Subregion suggests that their higher fertility (Table 1) may have been a means to ensure adequate family size: this pattern is most expected in high-mortality environments where child labor supported household survival (Tolnay 1995).

We further find that child mortality was positively associated with county malaria mortality rates among all mothers, but especially Black mothers (*Hypothesis 2;* see footnote 7). This finding is important in the context of Black farm women’s higher fertility rates (Table 1, Tolnay 1984); greater likelihood of fieldwork exposure (Brannen 1924); and poorer nutrition (Dirks and Duran 2001). High-fertility mothers give birth and nurse over longer child-bearing careers. Current bouts of severe caloric restriction and/or chronic malnutrition impairs children’s peri-natal growth, breastfeeding, and development trajectories (Woods 2009). In vicious cycles, chronic infectious diseases (malaria, TB) impair maternal-child nutrition while malnutrition increases susceptibility to disease and disease severity (Woods 2009). For example, bioarcheologists find linear enamel hypoplasia (LEH) or tooth enamel formation defects present – resulting from generalized stressors (malnutrition and/or disease) before age 6 – in about 80 percent of children buried in Arkansas Plantation Subregion Cedar Grove Cemetery (Franklin and Wilson 2020). Another indicator of poor nutrition, bone expansion around the eye orbit (cribra orbitalia) was present among 58 percent of children in this cemetery (Rose 1989). Evidence from the site found about half (58 percent) of child deaths occurred between ages one and five; deaths under five clustered at 18 months because weaning, too often, was followed by a protein-deficient diet that triggered rounds of diarrhea and infectious disease (Rose 1989).

In a third finding, Arkansas mothers living in counties with a greater proportion of tenant farms had higher child mortality (*Hypothesis 3*). That is, mothers’ rates were higher in counties where the household farm distribution skewed toward lower socioeconomic position (lower rungs of the Agricultural Ladder). Additionally, all mothers, but especially Black mothers living in counties with higher Black farm ownership rates (e.g., collective resources), had lower rates of child loss (*Hypothesis 4*; see Footnote 7).

Census data did not allow us to statistically pursue pathways from plantation life, disease environments, and Jim Crow-era tenancy to child mortality. We used oral accounts to ask: How was plantation life associated with health-related conditions of life for Black children during the Jim Crow era? What can we learn from witnesses who lived through this era? A first answer, among BTV participants with experience of plantation-mode tenancy, involved themes of work, hunger, housing, and racial prejudice. Participants described outdoor fieldwork early in life, often conflicting with schooling. They described watching mothers and/or becoming mothers who, despite arduous work, could not escape cycles of field work, hunger, and impoverishment with a need to take on additional paid work to feed families. They recalled exposure to discrimination and social disregard, including isolation from White public spaces: they described emotion-management amidst stresses of forced acquiescence to harsh conditions, upon threat of violence (Chafe et al. 1990; Jones 1992).

While southern Black and White women might be tenants on the Agricultural Ladder, they mostly lived “in separate worlds … that rarely intersected” (Jones 1988: 24). These separate worlds took form in childhood: White children stayed in school while Black children often could not, due to work. Our statistical analyses revealed that lower maternal education among Black women – a robust predictor of child survival – was associated with child mortality. Separate worlds continued through midlife: White as well as Black Arkansas tenant farm women did field work and lived in substandard housing while White tenants more likely left plantation life (Elman et al. 2020) and moved up the Agricultural Ladder (Alston and Ferrie 2005). Consequently, Black women tenants would remain on the lower rungs over longer, even whole portions, of their childbearing spans. The period between first birth to a last child’s departure from the home might span 35 to 40 years of life (Jones 1988).

Our one-state focus is a study limitation and yet an advantage. Historical demographic studies of child mortality based on national Census data necessarily trade off depth for breadth. They cannot parse whether or how specific places, ecologies, and local histories might shape child mortality. Small-area examinations of Place can capture such complexities. In the case of Arkansas, a Delta state whose promise of land ownership drew so many Black and White migrants (Elman et al. 2020; Gordon 1993; Matkin-Rawn 2014), we capture the peak (1910) of Black farm ownership rates (Hanson and Moneyhon 1989). Our findings suggest that the racial gap in child mortality was wider in southern states with lower Black farm ownership rates and/or more repressive economic and social regimes limiting “ability to rise.” Also, if a one-state analysis lacks breadth, it facilitates systematic use of qualitative reports. A main study strength is that, by adopting a mixed-methods approach, we can deeply explore structural racism, life experiences of those who survived it, and learn why so many children did not.

Another study limitation involves selective survival as the healthiest, most advantaged women survived to report oral histories. We found it useful to focus on a state, frontier Arkansas, where most women had high fertility. Fertility control, more common among White women in 1910, is associated with survival advantage (Hurt et al. 2006). The high fertility among all Arkansas women works to reduce racial bias in selective survival although selectivity remains; remaining selectivity works in the direction of our underestimating race differences in child mortality. Another limitation is that our dependent variable could not limit the observation of child deaths to given ages 1, 3, or 5. But a mixed-methods study (by definition) must also consider the substantive meaning of a dependent variable (child deaths). WPA and BTV accounts suggest that a child’s death, regardless of age, was the most salient fact for mothers.

Racial health disparities occur in the context of social-institutional fields whose elements combine differently across time and place (Williams and Mohammed 2013). The study of racial health disparities in separate times and places is, therefore, important. Historical study is also important because the health disparities that arose under racial regimes of the past were not contained in the past but continued into the present. A hidden intergenerational reproductive arm of own-mother birth weight, disease, and (mal)nutrition contributes to offspring reproductive and general health (Williams and Mohammed 2013). The health of children of high-risk mothers needs to be understood in light of the sociohistorical circumstances that produced the previous generation’s health disparities. More critically, structural racism, a fundamental cause of racial health disparities, does not remain buried in the past. As our study shows, socio-political regimes may change while structural forces persist, even if in shifting form, to shape racial health disparities going forward.

## Supplementary Material

Supplementary material

**Supplementary material.** The supplementary material for this article can be found at https://doi.org/10.1017/ssh.2025.15

## Figures and Tables

**Table 1.** Descriptive statistics: 1910 complete-count IPUMS, Arkansas

	All mothers	Black mothers	White mothers
Number of children ever born (Mean/SD)	4.11 (2.96)	4.24 (3.32)	4.05 (2.79)
Number of children surviving (Mean/SD)	3.09 (2.30)	2.88 (2.45)	3.18 (2.23)
Number of child deaths (Mean/SD)	1.02 (1.54)	1.36 (1.86)	0.88 (1.34)
Age (Mean/SD)	31.9 (8.45)	31.8 (8.41)	32.0 (8.5)
**Maternal characteristics/ resources (%)**			
Black mothers	30.4	–	–
Born in another state	42.3	45.3	41.0
Literate (can read and write)	86.2	71.2	92.8
Currently married	87.2	78.3	91.1
**Household rank (%)**			
Tenant farm household	31.8	47.4	24.9
Non-farm household	39.1	38.9	39.3
Owned farm household	29.1	13.6	35.8
Rural household	86.6	85.0	87.2
**Geographic subregion (%)**			
Plantation	38.8	75.0	23.0
Upcountry	23.3	1.8	32.7
Coastal plain and mixed	37.9	23.2	44.3
**Subregional groups (%)**			
Group 1: Upcountry	9.7	0.1	13.9
Group 2: Upcountry	13.6	1.7	18.8
Group 3: Coastal plain, mixed	14.2	1.7	19.7
Group 4: Coastal plain, mixed	15.8	10.3	18.2
Group 5: Coastal plain, mixed	7.9	11.2	6.4
Group 6: Plantation	3.4	3.3	3.5
Group 7: Plantation	21.3	34.8	15.4
Group 8: Plantation	14.1	36.9	4.2
**County-level household rank**			
County proportion tenant farms	0.51 (0.19)	0.64 (0.19)	0.45 (0.16)
County proportion black owned farms	0.07 (0.06)	0.12 (0.05)	0.05 (0.06)
**Health Environment**			
County malaria mortality rate (Mean/SD)	3.5 (3.32)	4.9 (2.9)	2.9 (3.3)
County population density (Mean/SD)	38.4 (24.3)	43.3 (25.7)	36.2 (23.4)
*N*	234,811	71,268	163,543

**Table 2.** Negative binomial count regressions: Child mortality rate, mothers ages 15–49

	IRR	99% CI	IRR	99% CI	IRR	99% CI
Black mothers^a^	1.49**	1.40–1.58	1.17**	1.11–1.24	1.19**	1.13–1.25
**Maternal resources**						
Age			1.01**	1.01–1.01	1.01**	1.01–1.01
Born in another state			1.07**	1.05–1.09	1.05**	1.03–1.06
Literate (read and write)			0.82**	0.79–0.84	0.83**	0.80–0.85
Currently married			0.92**	0.91–0.94	0.93**	0.91–0.94
Literate black mother			1.12**	1.08–1.16	1.10**	1.06–1.13
**Household rank**						
Tenant farm household			1.12**	1.09–1.14	1.09**	1.07–1.11
Tenant non-farm household			1.20**	1.17–1.22	1.18**	1.16–1.20
Rural household			1.09**	1.05–1.13	1.11**	1.08–1.15
**Geographic subregion**						
Group 1 upcountry			0.72**	0.66–0.78		
Group 2 upcountry			0.83**	0.76–0.92		
Group 3 coastal/mixed			0.94	0.88–1.00		
Group 4 coastal/mixed			0.84**	0.79–0.90		
Group 5 coastal/mixed			0.77**	0.70–0.84		
Group 6 plantation			0.98	0.86–1.12		
Group 7 plantation (ref: Group 8)			0.97	0.90–1.06		
**County-level household rank**						
Proportion Tenant Farms					1.67**	1.44–1.93
Proportion black owned farms					0.95**	0.93–0.97
**Health environment**						
Malaria Rate					1.03**	1.02–1.04
Population density					0.58*	0.40–0.83
Density squared					1.08**	1.03–1.13
Pearson Chi Sq. /df	0.84		0.87		–	
QIC	–		-		308980.1	
N	234,811		234,811		234,811	

a Predictive Margin (99%CI) of Race Variable: Model 1, Black .315** [.289–.339], White .211** [.200–.222], Difference .103** [.08–.127]. Model 2, Black .278** [.260–.297], White .216** [.207–.224], Difference .063** [.043–.083]. Model 3, Black .280** [.265–.295], White .215** [.208–.222], Difference .065** [.046–.083].

* *p* <.01

** *p* <.001 (two-tailed tests).

## Footnotes

1 Southern farm mechanization lagged (Jung 2020), in use of tractors (1935–1946, breaking land), mechanical cotton pickers (1948–1956, harvesting), and herbicides (post–1950, weeding); less than half of Mississippi Delta plantations had adopted tractor use in 1946 (Aiken 1998).

2 A frontier has a population density of between 2 and 6 persons per square mile. Arkansas briefly exited frontier status in 1860 but re-entered it by Civil War’s end as residents fled the state or died (Bolton 1998: 18; Moneyhon 1994).

3 In 1900, 92 percent of the Arkansas population was rural; less than 2 percent earned non-farm wages (Moneyhon 1997).

4 Two Black interviewers and the most experienced interviewer on the FWP project conducted Arkansas interviews (Cantrell 2004).

5 Interaction effects of Group and race did not improve model fit.

6 Other variable interactions with race did not meet a *p*<.01 threshold or improve model fit.

7 The malaria Average Marginal Effect (AME) was .008 (S.E. .001, *p*<.001) for Black women, .006 (S.E. .001, *p*<.001) for White women. The county proportion of tenant farms AME was .068 (S.E. .013, *p*<.001) for Black women, .053 (S.E. 0.009, *p*<.001) for White women. The county proportion of Black farms AME was −.014 (S.E., *p*<.001) for Black women, –.011 (S.E. *p*<.001) for White women.
