# Supplementary material for: ‘Little Baby’s gone to Heaven’: A Mixed-Methods Study of Black Children’s Survival Disadvantage in Jim Crow-Era Arkansas
Source: Soc Sci Hist. Author manuscript; Available in PMC 2025 Jul 17. (PMC12270450; doi:10.1017/ssh.2025.15)
Supplement: Supplementary material [file NIHMS2094357-supplement-Supplementary_material.pdf]

Online Supplementary Material

Data: *Plantation Farming in the U.S.* (1916)

We linked an Arkansas complete-count 1910 IPUMS sample to Arkansas county-level data, identifying Arkansas plantation counties, taken from Mandle (1992). Mandle (1992) lists the Arkansas counties enumerated in *Plantation Farming in the U.S.* (U.S. Census Bureau 1916). This Census was a special product: by the twentieth century's turn, the U.S. Census Bureau and Department of Agriculture recognized that different modes of agriculture—independent (single) farms and multi-farm plantations—operated in the South. They also were aware of a problem: they had been enumerating all farms in Decennial Censuses, whether independent or part of a multi-farm enterprise, as independent farms. Hence, they conducted a special Census in 1910 to enumerate plantations, defined as five or more farms that "operated as a single unit with respect to the methods of control of labor and products" (Brannen 1924: 9). On December 1, 1916, in his Letter of Transmittal for the Plantation Census, Sam L. Rogers, U.S. Census Director wrote:

> Because of the very great importance of the plantation system in the South, where are located nearly half the farms in the United States, this work was undertaken to obtain as much information as possible for use in connection with the study of rural life problems and the administration of the various congressional enactments for the benefit and extension of American agriculture.

The U.S. Census Bureau fielded this first plantation Census, along with the Thirteenth Decennial Census (1910) and published results, *Plantation Farming in the U.S.,* in 1916. While the Bureau did not publish the rules that they used to identify/select the plantation counties that they enumerated, they noted that they selected counties, at least partially, based on Black county population composition (proportion Black). The Bureau noted (Plantation Farming in the United States, 1916:16):

> it was known in advance that the plantation tenant system is for the most part confined to certain sections of the South, following primarily the sweep of the great cotton belt and of the belt containing a very large proportion of Negro population…In the great majority of the counties for which plantation statistics are presented the Negroes constituted at least half of the total population…there are comparatively few counties outside of the area for which plantation statistics are presented in which the proportion is as high as 50 percent.

Supplemental Figures 1 and 2 below illustrate the 1910 overlap between the spatial distribution of the Black population and plantation-style agricultural production in Arkansas. The state's population distribution (1910 county proportion Black) is shown in Supplemental Figure 1; geographic subregions are shown in Supplemental Figure 2.

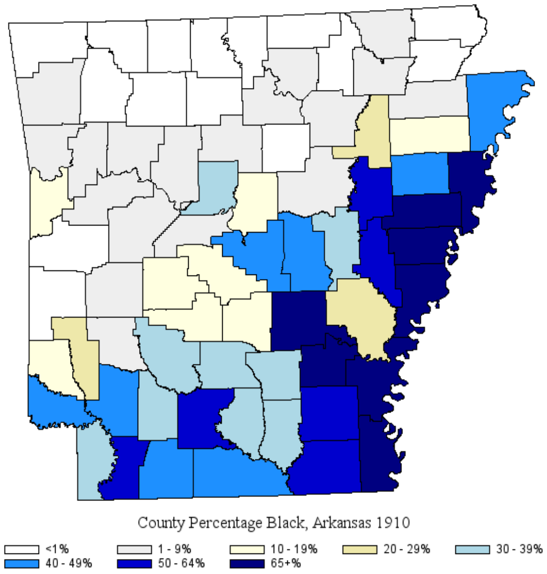

Supplement Figure 1. County Percentage Black, Arkansas 1910
Sources: Haines and ICPSR (2010); National Historical Geographic Information System (Manson et al. 2019).

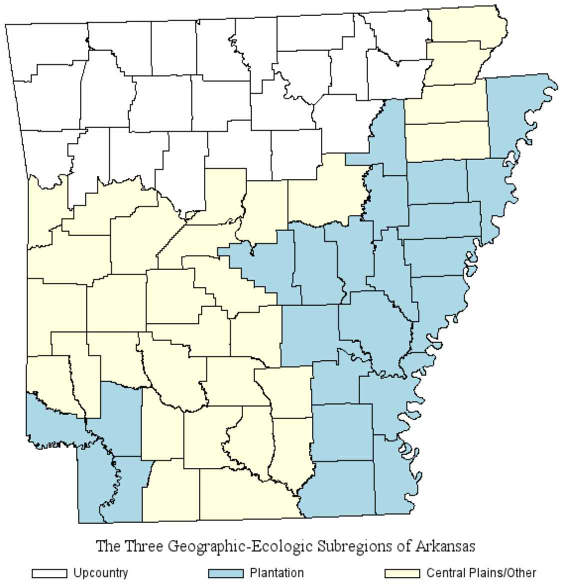

Supplement Figure 2. Arkansas Subregions, 1910
Sources: Plantation counties, Mandle (1992: Appendix); Upcountry counties, Blevins (2002: 5). National Historical Geographic Information System (Manson et al. 2019).

Arkansas had three geographic-ecologic subregions in 1910 (Supplement Figure 2), where: “a diagonal line drawn from the northeast corner of the state to the southwest corner” separated mountainous *Upcountry* from *Lowlands* (McNeilly 2000: 1, 2) with the latter further divided into a Mississippi River alluvial plain (Delta) bordering Tennessee and Mississippi, and a sandy-soiled Coastal Plain, bordering Louisiana and Texas. The Coastal Plain, northeast counties had limited large-scale production in 1910 (Whayne 1996).

Unfortunately, while the published report of *Plantation Farming in the U.S.* (1916) and its statistics aggregated at the state level are publicly available, the data collected by enumerators upon which the report is based, are not (Elman et al. 2015, 2019; Mandle 1992; Virts 1991, 2006). However, a U.S. Bureau of Agricultural Economics economist, C.O. Brannen, had access to the original collected *Plantation Farming in the U.S.* data; his publication, *Relation of Land Tenure to Plantation Organization* (1924) provides maps of enumerated Plantation Census states-counties. Economic historian Mandle (1992), utilizing Brannen (1924), listed 1916 Plantation Census counties in the states of Alabama, Arkansas, Georgia, Louisiana, Mississippi, North Carolina, and South Carolina in the Appendix of his book. We used his list to identify plantation counties in Arkansas. We cross-checked his list of counties for Arkansas with Brannen’s map (1924, Figure 2) containing Arkansas; we found no inconsistencies.

We attached Arkansas plantation county, Upcountry county, Central Plain dichotomous measures (and other contextual measures) to the complete count 1910 IPUMS using U.S. Census Bureau Federal Information Protection Standard or FIPS codes that uniquely identify each state and, within-state, each county.

References

Elman, Cheryl, Andrew S. London, and Robert A. McGuire (2015) “Fertility, Economic Development and Health in the Early Twentieth-Century U.S. South.” Journal of Interdisciplinary History 46:185-223. doi: 10.1162/JINH_a_00831

Elman, Cheryl, Robert A. McGuire and Andrew S. London (2019) “Disease, Plantation Development, and Race-Related Differences in Fertility in the Early Twentieth-Century American South.” American Journal of Sociology 124:1327–1371. https://doi.org/10.1086/702008

Blevins, Brooks (2002). *Hill Folks, A History of Arkansas: Ozarkers and their Image.* Chapel Hill: University of North Carolina Press.

Brannen, C.O. (1924). Relation of land tenure to plantation organization. Washington, D.C.: U.S. Department of Agriculture Bulletin No. 1269.

Haines, Michael R. and ICPSR (Inter-university Consortium for Political and Social Research) (2010) Historical, Demographic, Economic, and Social Data: The United States, 1790-2002. Ann Arbor, Mich. ICPSR [distributor]. https://doi.org/10.3886/ICPSR02896.v3

Mandle, Jay (1992) Not Slave, Not Free, The African American Economic Experience since the Civil War. Durham, NC.: Duke University Press.

McNeilly, Donald P. (2000). The Old South Frontier, Cotton Plantations and the Formation of Arkansas Society, 1819-1861. Fayetteville: University of Arkansas Press.

Manson, S., Schroeder, J., Van Riper, D., Ruggles, S., 2019. *IPUMS National Historical Geographic Information System: Version 14.0* [Database]. IPUMS, Minneapolis, MN. https://doi.org/10.18128/D050.V14.0.

U.S. Census Bureau (1916) Plantation Farming in the U.S. Washington, DC.

Virts, Nancy (1991). “The Efficiency of Southern Tenant Plantations, 1900–1945.” Journal of Economic History 51: 385–395. https://doi.org/10.1017/S0022050700039012

Virts, Nancy (2006) “Change in the Plantation System, 1910–1945.” Explorations in Economic History 43:153–76. https://doi.org/10.1016/j.eeh.2005.04.003

Whayne, Jeannie M. (1996) A New Plantation South: Land, Labor, and Federal Favor in Twentieth-Century Arkansas. Charlottesville: University of Virginia Press.

Methods

See Table 2. The general negative binomial model using an offset is as follows (Zwilling 2013):

$$Y \sim \text{Negative Binomial } (\mu = \lambda, \text{Var} = \lambda + \alpha\, \lambda^2)$$
$$\ln(\lambda) = \beta_0 + \beta_1 x_1 + \beta_2 x_2 + \ldots + \beta_p x_p + \ln t$$

The above equation can be transformed as follows:

$$\ln(\lambda / t) = \beta_0 + \beta_1 x_1 + \beta_2 x_2 + \ldots + \beta_p x_p$$

Where:

λ is the expected value (mean) of count Y (in this study, number of child deaths)

$x_1, \ldots, x_p$ indicate variables

$\beta_0, \ldots, \beta_p$ are population regression coefficients to be estimated for x variables

t is an offset indicating exposure (number of children born, logged)

Var is the variance

α is the estimated distribution (overdispersion) parameter.

Note: The offset (log of children ever born) is not itself parameterized in the equation.

Zwilling, Michael (2013) Negative Binomial Regression. The Mathematica Journal 15: 1-13.

Bibliographic Index (Oral History Interviews)

Abbreviations:
WPA Work Projects Administration / FWP Federal Writers' Project
BTV Behind the Veil

Rose Adway [FWP, age 76] interviewed by Bernice Bowden in Pine Bluff, Arkansas.

Sarah Anderson [FWP, age 78] interviewed by Bernice Bowdan in Pine Bluff, Arkansas.

Geraldine Davidson [BTV, born 1927] interviewed by Paul Ortiz in Fargo, Arkansas, 1995.

Drucilla Davis [FWP, age 73] interviewed by Bernice Bowden in Pine Bluff, Arkansas.
Dora Strong Dennis [BTV, born 1900] interviewed by Paul Ortiz in Fargo, Arkansas, July 19, 1995.

Georgia Ford [BTV, born 1928] interviewed by Paul Ortiz and Mausiki Stacey Scales in Forest City, Arkansas, July 19, 1995.

Le Ester Jones [BTV, born 1930] interviewed by Paul Ortiz in Brinkley, Arkansas, July 12, 1995.

Mattie Bell Lowe [BTV, born 1920] interviewed by Doris G. Dixon, Brinkley, Arkansas, July 11, 1995.

William Thomas Malone [BTV, born 1914] interviewed by Doris Dixon in Cotton Plant, Arkansas, July 21, 1995.

Katie Swanigan [BTV, born 1920] interviewed by Doris G. Dixon, Brinkley, Arkansas, July 13, 1995.

Laura Thornton [FWP, age 105] interviewed by Samuel S. Taylor, Little Rock, Arkansas.
Willie Clyde Tims [BTV, born 1922] interviewed by Paul Ortiz in Magnolia, Arkansas, July 20, 1995.

Elizabeth Tunage [BTV, born 1932] interviewed by Mausiki Stacey Scales, Brinkley, Arkansas, July 10, 1995.

Samella White [BTV, born 1929] interviewed by Paul Ortiz in Brinkley Arkansas, July 17, 1995.
Oliver Williams [BTV, born 1926] interviewed by Doris Dixon in Cotton Plant, Arkansas, July 21, 1995.

Delores Twillie Woods [BTV, born 1925] interviewed by Mausiki S. Scales in Forrest City, Arkansas, July 19, 1995.

Work Projects Administration (1936-38). Slave Narratives: A Folk History of Slavery in the United States from Interviews with Former Slaves: Volume II, Arkansas Narratives, Parts 1-7. https://www.gutenberg.org/ebooks/author/3906 (accessed April 5, 2024).

Behind the Veil: Documenting African-American Life in the Jim Crow South (Arkansas Tapes) (BTV) (1995). Center for Documentary Studies, Duke University. Held at David M. Rubinstein Rare Book and Manuscript Library, Duke University.
https://archives.lib.duke.edu/catalog/behindtheveil
